# Quantitative gait analysis as a method to assess mechanical hyperalgesia modulated by disease-modifying antirheumatoid drugs in the adjuvant-induced arthritic rat

**DOI:** 10.1186/ar2290

**Published:** 2007-09-11

**Authors:** Shabana Usman Simjee, Huma Jawed, Javeria Quadri, Sheikh Arshad Saeed

**Affiliations:** 1 HEJ Research Institute of Chemistry, International Centre for Chemical and Biological Sciences, University of Karachi, Karachi 75270, Pakistan; 2 Dr Panjwani Centre for Molecular Medicine and Drug Research, International Centre for Chemical and Biological Sciences, University of Karachi, Karachi 75270, Pakistan

## Abstract

In the present study, azothioprine, chloroquine, D-penicillamine, methotrexate and sodium aurothiomalate (gold salt) were evaluated for possible disease-modifying effects in the adjuvant-induced arthritis model of human rheumatoid arthritis in rats. Gait analysis was used to examine the role of disease-modifying antirheumatic drugs in the development of pain. Body weights were also measured to monitor the progression of disease and the systemic antiarthritic effects of the test compounds used in this study, as well as their systemic toxicity. Our results showed that azothioprine (5 mg/kg/day), chloroquine (12.5 mg/kg/day), sodium aurothiomalate (2.5 mg/kg/day) and methotrexate (1 mg/kg/week) not only inhibited the macroscopic changes such as erythema and swelling of limbs, but also exhibited significant reversal of gait deficits seen in the untreated or saline-treated arthritic rats. No reduction in the body weights were observed in the arthritic rats treated with azothioprine, chloroquine, sodium aurothiomalate and methotrexate. D-Penicillamine (12.5 mg/kg/day), however, showed a significant reduction (*P *< 0.03) in the body weights of the arthritic rats over a period of 22 days; furthermore, it was unable to show any reduction in arthritic score (*P *< 0.1). In earlier experiments, chloroquine and methotrexate failed to suppress carageenan-induced edema, suggesting that the mode of antiarthritic action may be different from those of nonsteroidal anti-inflammatory agents. Since these disease-modifying antirheumatic drugs are reported to have an immunomodulatory role, especially the gold salt, which influences the monocyte–macrophage system, it is suggested that the observed antiarthritic effects of disease-modifying antirheumatic drugs may be partly attributed to their immunomodulatory activity.

## Introduction

Chronic pain, a devastating and widespread problem, is a syndrome that cuts across traditionally defined disciplinary boundaries within the health sciences. Patients with chronic pain, compared with all other medical conditions, suffer dramatic reductions in physical, psychological and social wellbeing [1-3]. Within this group of patients, arthritis is the second largest self-reported cause of pain [4]. Although there is no rheumatoid arthritis cure, there are effective treatments that can alleviate the symptoms and improve the quality of life. The nonsteroidal anti-inflammatory drugs and glucocorticoids are largely used for treatment of rheumatoid arthritis in spite of their systemic, gastric and renal toxicities [5-11]. These currently available analgesic and anti-inflammatory drugs are clearly not adequate therapy. In addition to these classical available therapies, there are several reports regarding the use of disease-modifying antirheumatic drugs (DMARDs), which act as potentially effective therapies for rheumatoid arthritis [12,13]. DMARD treatment is currently based on symptomatic relief of pain and inflammation associated with arthritis to increase joint function and mobility.

In order to study the effects of DMARDs in the progression of disease, we have used the adjuvant-induced arthritis model in the rat. This model has biochemical and pathological features similar to rheumatic disease in human, and merits investigation [14-19]. In this model, disease is characterized by joint pain, joint stiffness, joint swelling and tenderness, and muscle wasting leading to weight loss [20-22]. The aim of the present study was to identify the antiarthritic and antinociceptive effects of the DMARDs azothioprine (AZ), chloroquine (CQ), D-penicillamine (D-PEN), sodium aurothiomalate (gold salt (GS)) and methotrexate (MTX), and to measure any effect of these drugs on gait. Gait analysis allows highly sensitive, noninvasive detection and evaluation of many pathophysiological features, such as those occurring in Alzheimer's disease, arthritis, pain, Parkinson's disease, neuromuscular and skeletal muscle diseases. In addition, the method of gait analysis showed good evidence of reproducibility and reliability in our earlier studies [23,24]. It is suggested that changes in gait may be considered a potential marker of chronic pain.

## Materials and methods

### Animals

Female Sprague–Dawley rats weighing 215–230 g (8–10 weeks) were used in the study. The animals were kept at 21 ± 2°C on a 12-hour light/dark cycle with free access to standard laboratory rat food pellets and water. The ethical guidelines of the International Association for the Study of Pain in conscious animals were followed [25]. Rats were randomly distributed to each treatment group of six animals. The group size was determined as the minimum number of rats for valid statistical analyses based on a pilot study by our group. The group size of six has an 80% power to detect differences in the means.

### Induction of arthritis

Lyophilized *Mycobacterium tuberculosis *H37Ra (MT H37Ra; DIFCO Laboratories, Detroit, MI, USA) was used as an adjuvant to induce arthritis. Fresh adjuvant was prepared on the same day as arthritis was induced. A volume of 0.1 ml of a 1 mg suspension of MT H37Ra was injected intradermally at the base of the tail using a sterile hypodermic needle under anesthesia with a combination of ketamine/xylazine in the dose of 20 mg/kg/5 mg/kg (intraperitoneal). Treatment was initiated on the same day of arthritis induction.

### Drugs

The reference drug indomethacine and the DMARDs (AZ, CQ, D-PEN, GS and MTX) were purchased from Sigma Chemical Company (St Louis, MO, USA). The vehicle (saline) and drugs were administered intraperitoneally – except for GS, which was administered subcutaneously. The doses of drugs used in the present study were selected by perusal of the literature and preliminary dose-finding studies to obtain regimens that had no effect on gait in nonarthritic rats. In addition, concurrent test control rats were administered only with saline.

### Clinical assessment of collagen-induced arthritis

Rats were evaluated on alternate days for arthritis using a macroscopic scoring system, where 0 = no signs of arthritis, 1 = swelling and/or redness of the paw or one digit, 2 = two joints involved, 3 = more than two joints involved, and 4 = severe arthritis of the entire paw and all digits [26,27]. The arthritis severity score for each rat was calculated by adding the scores for each individual paw.

### Measurement of hind paw hyperalgesia and edema

The method for measuring hyperalgesia has been described previously [28]. The tendency of normal (naive), control and arthritic rats to vocalize following flexion of the tarsotibial joints of both hind paws was tested daily for 22 days starting from day 0. Hyperalgesia is reported as the mean ± standard error of the mean number of vocalizations following five flexions of the hind limb tarsotibial joints, considering maximal hyperalgesia (value = 1) when five vocalizations were obtained following five flexions of the paws.

The clinical severity of arthritis was also determined by quantitating the change in the paw volume (as an indicator of edema) with a plethysmometer (model 7140; Ugo Basile, Varese, Italy) following the hyperalgesia test. The advantage of using this method over diameter measurements of tibiotarsal joint is that it measures the limb in three dimensions and therefore takes into account any variability of the pattern of swelling of individual limbs. The volume of a hind paw is reported as the mean ± standard error of the mean in milliliters. All measurements were made at the same time of day. The body weight and hind paw volumes were measured in both the control and test groups on days 0 and then on alternate days until day 22 when the experiment ended.

### Gait analysis

Locomotion was recorded in test and control groups at the beginning of an experiment and was used as the baseline reading (day 0). The apparatus used for this purpose was the TreadScan system (Clever Sys. Inc., Reston, VA, USA). This system records a video of an animal (mouse or rat) running on a transparent treadmill as the input. A mirror is placed at an angle of 45° below the belt section of the chamber, which allows the viewing of floor/paw contacts. The video essentially captures the footprints of the animal during exercises on the treadmill. The software provided with this system (TreadScan) can analyze the video, and can determine various characteristic parameters that are related to the pathophysiological conditions. The parameters measured in this study include the stance time (paw in contact with the floor), the swing time (paw in the air), the stride length and the running speed.

### Statistical analysis

Statistical Package for the Social Sciences software (SPSS Inc. Chicago, IL, USA) was used to analyze the data. Throughout the study, the mean ± standard error of means was used to describe the data in the figures. The data were analyzed using two-way analysis of variance. Bonferroni's post-hoc test was used to determine which group means differ. With this test the SPSS Inc. software automatically adjusts the significant level for the multiple comparisons to avoid spurious significant differences being identified (any values <0.05 were considered significant). The Mann–Whitney U test was used for nonparametric data (inflammation scores).

## Results

### Effects of DMARDs on clinical signs of adjuvant-induced arthritis

**Figure 1:**
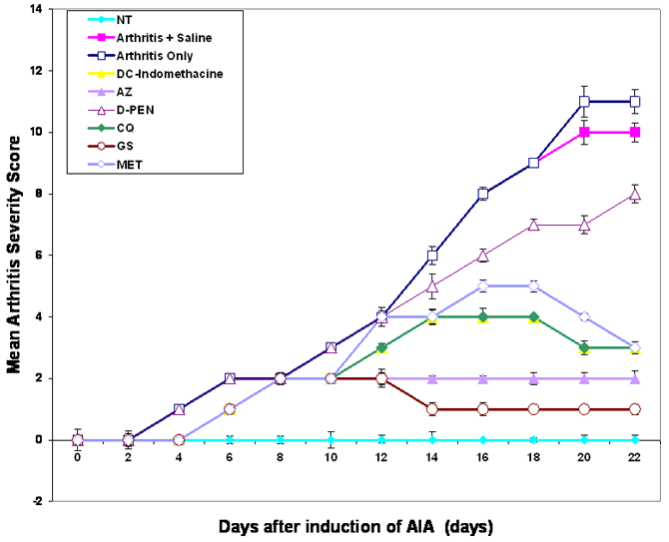
Arthritis severity scores in rats during the development of adjuvant-induced arthritis. Effect of azothioprine (AZ), chloroquine (CQ), D-penicillamine (D-PEN), sodium aurothiomalate (gold salt (GS)) and methotrexate (MTX) on the time course of the development and progression of arthritis, shown as the arthritis severity scores measured over a period of 22 days. Results are the mean ± standard error for six animals in each group. AIA, adjuvant-induced arthritis; DC, drug control; NT, no treatment.

**Figure 2:**
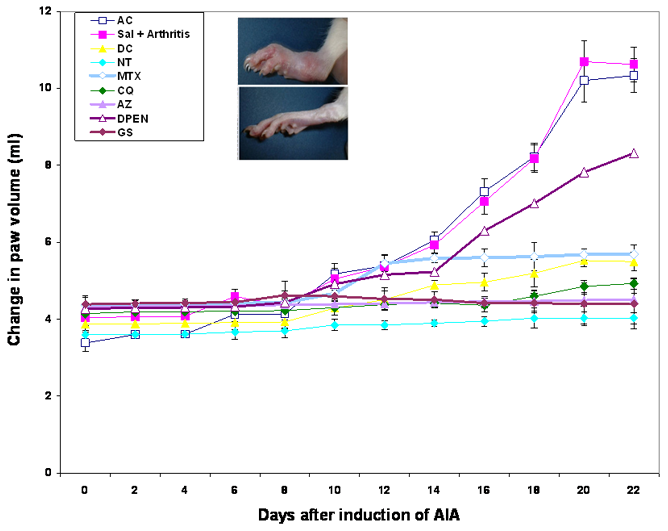
Time course of the change in hind paw volume after the induction of arthritis. Rats in all arthritic groups showed an increase in their paw volume until day 8; however, this increase in the azothioprine (AZ)-treated, chloroquine (CQ)-treated and sodium aurothiomalate (gold salt (GS))-treated arthritic rats was not significantly different from the normal control rats, and also no further increase in the paw volume was noticed in these groups. In contrast, arthritic rats treated with methotrexate (MTX) and indomethacine showed a gradual increase in their paw volume over a period of 22 days, but this increase was nonsignificant compared with normal control rats. The arthritic rats treated with D-penicillamine (D-PEN) or saline showed a significant increase in their paw volume over the period of 22 days (*P *< 0.006). Inbox pictures, difference between the arthritic rat paw and the normal rat paw. Results expressed as mean ± standard error (*n *= 6). AC, arthritic control; AIA, adjuvant-induced arthritis; DC, drug control; NT, no treatment; Sal + Art, saline and arthritis.

After day 10, animals began to show evidence of clinical inflammation in one or both hind paws. The first manifestation of disease was erythema of one or more ankle joints, followed by involvement of the metatarsal and interphalangeal joints. The typical time course for the development and progression of disease, as assessed by the mean arthritis severity score and the paw volume, is shown in Figures 1 and 2. Signs of an arthritic score of 3 in untreated arthritic rats, in arthritic rats treated with saline only or in arthritic rats treated with D-PEN were evident at day 10. The arthritic rats treated with AZ, CQ, GS, MTX and indomethacine, however, showed a score of 2 on day 10. The disease was progressive, with joint recruitment following the same pattern: tarsal, metatarsophalangeal and then interphalangeal. In the vehicle and nontreated arthritic group, the incidence of disease was 100% (that is, all animals in the group were affected) at day 12, and remained as such throughout the duration of the experiment. In contrast, treatment with indomethacine, AZ, CQ, GS and MTX exerted a significant attenuation in the incidence of adjuvant-induced arthritis: 80% with GS treatment (*P *< 0.02), 70% with AZ treatment (*P *< 0.05), 60% with CQ and indomethacine treatment (*P *< 0.05) and 50% with MTX treatment (*P *< 0.05). Hyperalgesia, the increase in vocalization in response to forced flexion of the tarsotibial joints, was evident from day 11 in arthritic animals, reaching a maximum value by the end of the experiment. Once the treatment was started, the animals in the arthritic groups treated with AZ, with CQ, with GS, with MTX and with indomethacine showed a marked decrease in the vocalization compared with their concurrent arthritic control animals.

A large increase was observed in the hind paw volume of untreated, saline-treated and D-PEN-treated arthritic rats compared with nonarthritic rats. Analysis of variance performed on the overall data showed that this increase in the paw volumes became significantly different from that of nonarthritic rats from day 10 onwards (*P *< 0.006). It was observed that the arthritic rats treated with AZ, with CQ and with GS showed a slight nonsignificant increase in their paw volume compared with normal control rats from day 8 to day 12. In contrast, arthritic rats treated with MTX and indomethacine showed a gradual but also nonsignificant increase in their paw volumes until the end of the experiment.

**Figure 3:**
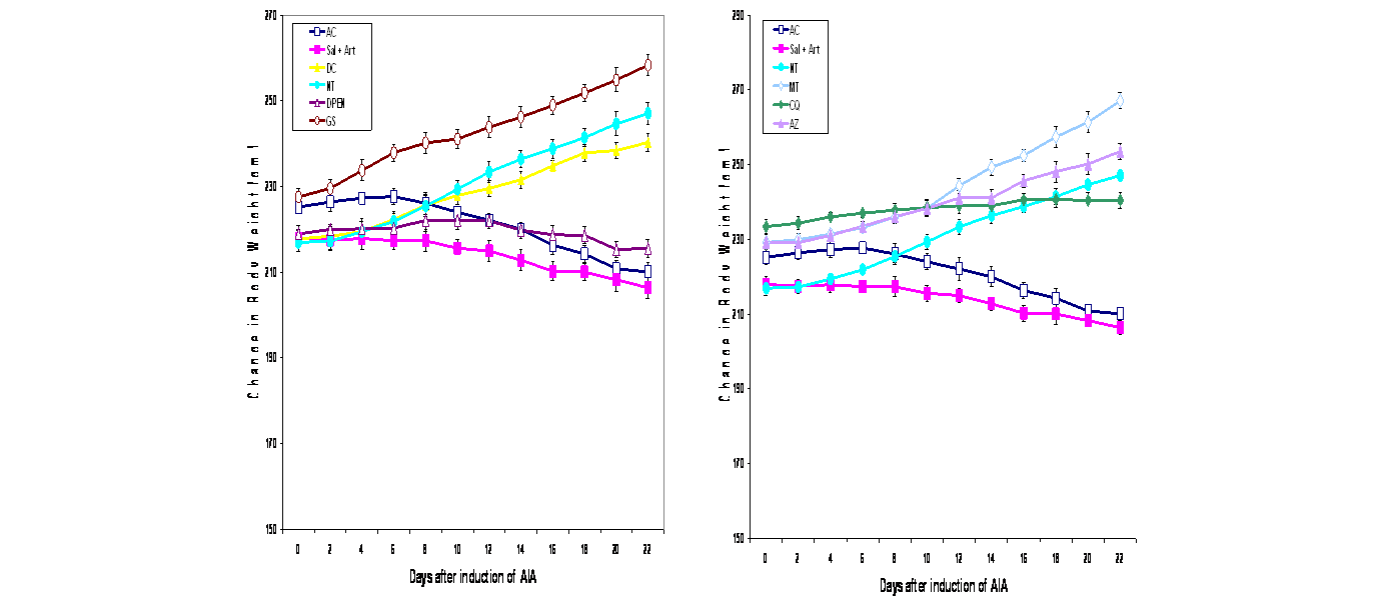
Time course of body weight changes in rats with adjuvant-induced arthritis. Effect of disease-modifying antirheumatic drugs (DMARDs) on the body weight change of the arthritic rats, measured over a period of 22 days. **(a) **Effect of D-penicillamine (D-PEN) and sodium aurothiomalate (gold salt (GS)) on body weights of adjuvant-induced arthritis (AIA) rats. **(b) **Effect of azothioprine (AZ), chloroquine (CQ) and methotrexate (MTX) on body weights of AIA rats. Results are the mean ± standard error for six animals per group. AC, arthritic control; DC, drug control; NT, no treatment; Sal + Art, saline and arthritis.

The body weights of the tested animals were not significantly different between the groups before commencement of the study. In the first 6 days the increment in body weight was similar in all groups and no significant differences were seen between them. After day 8, however, a gradual loss in body weight was observed that become significant on day 10 for untreated, saline-treated and D-PEN-treated arthritic rats as compared with the normal control rats. This weight loss was consistent until the end of the study (Figure 3). In contrast, the nonarthritic rats or the arthritic rats treated with AZ, with CQ, with GS, with MTX and with indomethacine showed no reduction in their body weights. The Bonferroni's post-hoc test for the individual time period showed that the significant difference in the mean body weight change of untreated arthritic rats and saline-treated nonarthritic rats started from day 10 to day 22 (*P *< 0.04). No significant difference, however, was observed within arthritic groups treated either with AZ, with CQ, with GS, with MTX and with indomethacine.

### Gait parameters

**Figure 4:**
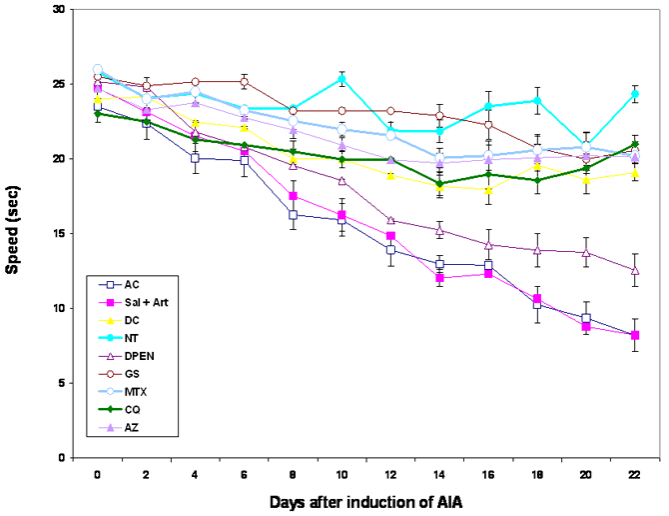
Time course of walking speed changes after the induction of arthritis compared with normal rats. Treated arthritic rats or untreated arthritic rats showed a gradual decrease in velocity from day 2 onwards. Arthritic rats treated with azothioprine (AZ), chloroquine (CQ), sodium aurothiomalate (gold salt (GS)), methotrexate (MTX) and indomethacine, however, exhibited a reversal of the deficit in velocity seen in the untreated rats or saline-treated arthritic rats from day 12 onwards. Results are the mean ± standard error (*n *= 6). AC, arthritic control; AIA, adjuvant-induced arthritis; DC, drug control; NT, no treatment; Sal + Art, saline and arthritis.

The speed or velocity of the nonarthritic rat showed little variation over the 22-day experiment, and was unaffected by drug treatment (Figure 4). Arthritic rats untreated or treated with saline or D-PEN showed a gradual decrease in their velocity from day 2 onwards. This was statistically significant on day 6 (*P *< 0.004). The AZ, CQ, MTX and indomethacine treatments of the arthritic rats showed a decrease in their speed from day 2 to day 12, but this decrease in the speed was not significantly different from the normal control rats. The arthritic rats treated with GS showed a slight nonsignificant decrease in their velocity from day 8.

The treatments with AZ, with CQ, with GS, with MTX and with indomethacine given to the arthritic rats showed a smaller decrease in the stride length from day 2 to day 6 (Table 1); thereafter, animals exhibited a reversal in the stride length compared with the untreated arthritic rats or the D-PEN-treated arthritic rats, so much so that the stride lengths were indistinguishable from normal rats from day 16 onwards. Arthritic rats untreated or treated with saline or D-PEN showed a steady reduction in their stride length over a period of 22 days. This reduction in the stride length became significant from day 10 onwards when compared with normal control rats (*P *< 0.01). Table 1 compares the day 0 values with the day 22 values to illustrate these effects. Arthritis also caused an increase in the stance time and the swing time from day 4 onwards. Administration of GS and of indomethacine, however, significantly decreased the gait deficits seen in untreated or saline-treated arthritic rats from day 6 onwards (*P *< 0.003). The arthritic rats treated with AZ, with CQ and with MTX were able to show a significant reversal in these gait parameters only after day 16 (*P *< 0.05 for stance time and P < 0.02 for swing time) when compared with untreated arthritic rats.

**Table 1:** Effects of azothioprine, chloroquine, D-penicillamine, sodium aurothiomalate (gold salt) and methotrexate administration over 22 days on gait parameters of arthritic rats

	Day	Stride length (cm)	Stance time (s)	Swing time (s)
Normal control rats	0	14.44 ± 0.40	5.512 ± 0.05	5.842 ± 0.09
	22	14.5 ± 0.34	5.53 ± 0.13	6.011 ± 0.87
Arthritic rats				
Vehicle (saline)	0	15.33 ± 1.05	5.06 ± 0.01	7.12 ± 1.01
	22	6.52 ± 0.57	16.82 ± 0.08	15.32 ± 0.43
Azothioprine (5 mg/kg/day)	0	13.66 ± 0.29	5.97 ± 0.110	7.24 ± 0.079
	22	13.16 ± 0.50	7.01 ± 0.091	6.57 ± 0.11
Chloroquine (12.5 mg/kg/day)	0	14.28 ± 0.49	6.11 ± 0.33	6.82 ± 0.523
	22	14.01 ± 0.48	6.98 ± 0.055	7.51 ± 0.073
D-Penicillamine (12.5 mg/kg/day)	0	14.95 ± 0.29	4.21 ± 0.017	6.54 ± 0.15
	22	8.59 ± 0.31	11.5 ± 0.091	10.01 ± 0.11
Gold Salt (GS, 2.5 mg/kg/day)	0	15.89 ± 0.49	5.78 ± 0.33	6.72 ± 0.48
	22	14.98 ± 0.44	6.07 ± 0.055	5.98 ± 0.093
Methotrexate (0.5 mg/kg/week)	0	16.01 ± 0.29	5.59 ± 0.017	5.83 ± 0.075
	22	12.79 ± 0.50	7.23 ± 0.09	7.21 ± 0.121
Indomethacine(5 mg/kg/day)	0	14.22 ± 0.49	4.85 ± 0.33	6.07 ± 0.077
	22	14.01 ± 0.48	7.17 ± 0.055	8.22 ± 0.23

Results expressed as the mean ± standard error (*n *= 6) on day 0 and day 22 of the experiment.

## Discussion

The treatment of rheumatoid arthritis has gone through many major changes in the past. The concept that drugs should be used to slow down damage caused by the disease rather than simply to control symptoms resulted in various agents being introduced, which were initially called 'slow-acting antirheumatic drugs' and were later referred to as DMARDs. These DMARDs are reported to be widely used in treating rheumatoid arthritis in humans [29-34]. The goal of DMARDs is the remission or control of inflammatory joint disease, such as rheumatoid arthritis. While other arthritis medicines attack symptoms such as inflammation, DMARDs actually treat the disease.

It has been reported that DMARDs such as AZ, CQ, MTX and GS play an important role in slowing the progression of disease in patients with various autoimmune disorders. It has also been suggested that low-dose AZ, CQ, GS and MTX in treatment for adjuvant-induced arthritis seems to exert anti-inflammatory effects by acting at different levels of the pathophysiological cascade. AZ, CQ, GS and MTX have been shown to inhibit T-cell responses during inflammatory reactions [35-37]. Both GS and MTX were found to inhibit function of phagocytic cells and both monocytic/lymphocytic proinflammatory cytokines involved in rheumatoid synovitis, and this seems to be the key role in the sustained anti-inflammatory actions exerted by low-dose MTX [38-48].

In the present study we describe a possible novel approach to quantify the hyperalgesia in a rat model of rheumatoid arthritis using gait analysis. It has previously been reported that gait changes in the arthritic rat can be used as an objective measure of chronic pain [23,49]. The principal aim of this study was to examine the effect of prolonged administration of low doses of AZ, CQ, D-PEN, GS and MTX on the progression of hind paw inflammation, disease progression and gait analysis. We found a strong correlation between parameters obtained from gait analysis and the disease progression in the adjuvant-induced arthritic rats. The administration of AZ, CQ, GS and MTX doses used in this study that had no effect on gait in normal rats produced a complete reversal of the gait deficits seen in the untreated, saline-treated and D-PEN-treated arthritic rats. This suggests that a prolonged administration of low doses of these DMARDs is effective in preventing the development of chronic pain, which, once established, is difficult to treat with conventional analgesics. These DMARDs also had a significant effect on disease progression, measured by no further weight loss as observed in the untreated or saline-treated adjuvant-induced arthritis. The DMARDs also significantly reduced the joint inflammation when administered for a prolonged time (over 22 days in case of our study) at low doses.

## Conclusion

We demonstrate here that continuous administration of DMARDs at low doses not only reduces the inflammation as seen from the macroscopic studies of the arthritic paws, but also modulates the mechanical hyperalgesia in treated arthritic rats. In addition, the method of gait analysis showed good evidence of reproducibility in the present study, and our data suggest that the changes in gait may be considered a potential marker of chronic pain in arthritic rats.

## Abbreviations

AZ = azothioprine; CQ = chloroquine; D-PEN = D-penicillamine; DMARD = disease-modifying antirheumatic drug; GS = sodium aurothiomalate (gold salt); MTX = methotrexate.

## Competing interests

The authors declare that they have no competing interests.

## Authors' contributions

All four authors participated in the study. SAS conceived of the study and participated in coordination. SUS designed the study, drafted the manuscript and performed the analysis of data. HJ participated in the study and performed the gait analysis. JQ is a graduate student who provided us the drugs/compounds used in the study. All authors read and approved the final manuscript.
